# Native T1 mapping for the diagnosis of cardiac amyloidosis in patients with left ventricular hypertrophy

**DOI:** 10.1007/s00392-022-02005-2

**Published:** 2022-03-31

**Authors:** Daniel Lavall, Nicola H. Vosshage, Romy Geßner, Stephan Stöbe, Sebastian Ebel, Timm Denecke, Andreas Hagendorff, Ulrich Laufs

**Affiliations:** 1 Klinik und Poliklinik für Kardiologie, Universitätsklinikum Leipzig, Liebigstrasse 20, 04103 Leipzig, Germany; 2 Klinik und Poliklinik für Diagnostische und Interventionelle Radiologie, Universitätsklinikum Leipzig, Liebigstrasse 20, 04103 Leipzig, Germany

**Keywords:** Left ventricular hypertrophy, Cardiac amyloidosis, Cardiac magnetic resonance, Native T1 mapping

## Abstract

**Background:**

Cardiac magnetic resonance (CMR) with parametric mapping can improve the characterization of myocardial tissue. We studied the diagnostic value of native T1 mapping to detect cardiac amyloidosis in patients with left ventricular (LV) hypertrophy.

**Methods:**

One hundred twenty-five patients with increased LV wall thickness (≥ 12 mm end-diastole) who received clinical CMR in a 3 T scanner between 2017 and 2020 were included. 31 subjects without structural heart disease served as controls. Native T1 was measured as global mean value from 3 LV short axis slices. The study was registered at German clinical trial registry (DRKS00022048).

**Results:**

Mean age of the patients was 66 ± 14 years, 83% were males. CA was present in 24 patients, 21 patients had hypertrophic cardiomyopathy (HCM), 80 patients suffered from hypertensive heart disease (HHD). Native T1 times were higher in patients with CA (1409 ± 59 ms, *p* < 0.0001) compared to healthy controls (1225 ± 21 ms), HCM (1266 ± 44 ms) and HHD (1257 ± 41 ms). HCM and HHD patients did not differ in their native T1 times but were increased compared to control (*p* < 0.01). ROC analysis of native T1 demonstrated an area under the curve for the detection of CA vs. HCM and HHD of 0.9938 (*p* < 0.0001), which was higher than that of extracellular volume (0.9876) or quantitative late gadolinium enhancement (0.9406; both *p* < 0.0001). The optimal cut-off value of native T1 to diagnose CA was 1341 ms (sensitivity 100%, specificity 97%).

**Conclusion:**

Non-contrast CMR imaging with native T1 mapping provides high diagnostic accuracy to diagnose cardiac amyloidosis in patients with left ventricular hypertrophy.

**Supplementary Information:**

The online version contains supplementary material available at 10.1007/s00392-022-02005-2.

## Background

Cardiac amyloidosis (CA) in an underdiagnosed infiltrative disorder characterized by the aggregation of misfolded amyloid fibrils in the myocardium with significant impact on morbidity and mortality. The two most common forms of CA are transthyretin (ATTR) and light-chain (AL) amyloidosis. ATTR amyloidosis is a disease of the elderly, its prevalence increases with age. AL amyloidosis is a complication of plasma cell dyscrasia [1–3]. Since there are no specific symptoms of CA, the diagnosis is challenging and often delayed to advanced stages of the disease. This precludes early treatment to inhibit disease progression effectively. Therefore, there is a clinical need to improve the diagnosis of CA. According to the current recommendations, the diagnosis of CA is made by either pathologic bone scintigraphy (the radioactive tracer binds to amyloid) and blood test for plasma cell dyscrasia, or by endomyocardial biopsy [1, 3–5].

CA is characterized by increased left ventricular (LV) wall thickness. However, increased LV wall thickness is common in the elderly population. Frequent causes are arterial hypertension with hypertensive heart disease (HHD) and aortic stenosis. While aortic stenosis is usually diagnosed by echocardiography, HHD needs to be dissected from less common causes of increased LV wall thickness, such as hypertrophic cardiomyopathy (HCM) and cardiac amyloidosis (CA) [6].

Cardiac magnetic resonance (CMR) tomography characterizes cardiac size, function and morphology. The application of Gadolinium-based contrast is regularly used to detect myocardial scar and interstitial remodeling. The pattern of Late Gadolinium Enhancement (LGE) is characteristic for specific cardiomyopathies and reflects prognosis [7, 8]. Mapping techniques use the relaxation properties of the myocardium for tissue characterization [9]. CA is characterized by increased LV mass, diffuse or circular subendocardial LGE, elevated native T1 values and increased extracellular volume fraction (ECV) [1, 10]. Native T1 mapping has been demonstrated to discriminate CA from non-CA cardiomyopathies in selected patients (9). The role of native T1 in unselected patients with increased LV wall thickness is currently unknown.

The aim of this study was (1) to evaluate the role of native T1 mapping for the differential diagnosis of increased LV wall thickness and (2) to identify specific cut-off values for native T1 time in 3 T CMR to diagnose CA.

## Methods

### Patient population

**Fig. 1:**
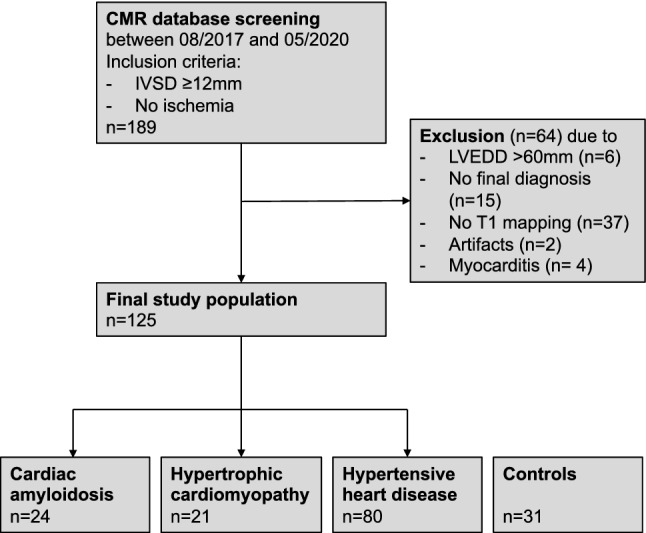
Study flow chart. Screening revealed 189 eligible patients. 64 patients were removed for prespecified exclusion criteria. Thus, 125 patients were included in the final analysis. 31 healthy persons served as control groups to establish the reference mapping values. *IVSD* inter-ventricular septum thickness at end-diastole; *LVEDD* left ventricular end-diastolic diameter

Patients with increased LV wall thickness (≥ 12 mm end-diastole) without myocardial ischemia who were studied on CMR for clinical indication between 08/2017 and 05/2020 were included in the study. Data derived from the institutional CMR database of the University Hospital Leipzig. The study flow chart is shown in Fig. 1. Patients with myocardial ischemia on perfusion stress CMR were excluded to avoid interaction of significant coronary artery disease with T1 relaxation times. Patients with clinically suspected or confirmed myocarditis (because myocardial inflammation increases T1 values; *n* = 4), LV dilatation > 60 mm end-diastolic diameter (because of advanced structural remodeling; *n* = 6), no final diagnosis (*n* = 15), without T1 mapping (*n* = 37) or artifacts (precluding mapping analysis in > 3 myocardial segments; *n* = 2) were excluded. In the final analysis, 24 patients with CA (18 with ATTR, 5 with AL, 1 undetermined), 21 patients with HCM and 80 patients with HHD were included. HCM was defined as increased LV wall thickness on echocardiography or CMR without sufficient explanation by abnormal loading conditions, according to the ESC guideline [6]. Late Gadolinium Enhancement with a patchy mid-wall pattern in the areas of pronounced hypertrophy or the right ventricular insertion points supported the diagnosis of HCM [6, 12]. In patients with HCM and concomitant arterial hypertension, the extent of blood pressure augmentation did not explain the increased LV wall thickness sufficiently according to the attending physician and the study data reviewing board. HHD was established in patients with increased LV wall thickness and arterial hypertension without LGE findings suggestive of CA or HCM and without significant aortic stenosis (> moderate). Endomyocardial biopsy was performed if non-invasive imaging was inconclusive. CA was diagnosed by either tissue histology (*n* = 19) or bone scintigraphy and blood test for plasma cell dyscrasia [5, 13]. The physician who performed clinical CMR had full access to the patient data management system of the hospital. 31 healthy subjects without evidence of apparent structural heart disease on CMR served as control group.

The institutional review board (ethics committee of Leipzig University) approved the study (no. 176/20-ek). Informed consent was not required for this retrospective study. The study was registered at the German clinical trial registry (www.drks.de), study ID DRKS00022048.

### CMR protocol and analysis

CMR was performed using a Philipps Achieva 3.0 Tesla (Philips Healthcare) clinical scanner. The sequence protocol was prefaced of survey images in coronal, sagittal and transversal orientation. Retrospectively gated steady state free precession cine images (SSFP) were acquired in long and short axis orientation of the LV (repetition time (TR), 45 ms; echo time (TE), 1.4 ms; flip angle (FA), 45°; voxel size, 1.7 × 1.7 x 6 mm^3^). Native and post-contrast T1 mapping in basal, mid and apical short axis slices of the LV was measured using the modified look-locker inversion recovery sequence (MOLLI; TR, 2.1 ms; TE, 0.98 ms; FA, 20°; voxel size, 2.0 × 2.0 × 10.0 mm^3^). Late gadolinium Enhancement (LGE) was assessed at least 10 min after intravenous application of 0.15 mmol/kg body weight Gadobutrol (Gadovist^®^, Bayer) in a short axis stack using phase-sensitive inversion recovery sequence (PSIR) (typical image parameters were: TR, 3.53 ms; TE, 956 ms; FoV, 300 mm; matrix, 256 mm; voxel size, 0.6 × 0.6 × 10 mm^3^).

Data were analyzed offline using the Intellispace software (Philips Healthcare) according to the current recommendations [14]. End-diastolic LV diameters and inter-ventricular septal wall thickness were measured in a mid-ventricular short axis. LV volumes, mass, stroke volume (SV) and ejection fraction (EF) were determined in short axis covering the entire LV. Papillary muscles were subtracted from blood cavity. LGE was measured quantitatively in a short axis LV stack (PSIR), whose orientation was identical to the LV short axis cine sequences. Based on the endocardial and epicardial border contours for LV mass quantification, LGE was detected semi-automatically and corrected manually if necessary.

T1 mapping was measured as a global T1 value from 3 short axis slices (basal, mid and apical) each containing 6 segments before contrast administration. Since the study tested whether non-contrast CMR with native T1 mapping would be appropriate to diagnose CA, mapping was analyzed without the corrections for the presence of LGE. For ECV calculation, regions of interest from native and post-contrast T1 mapping were used after review and correction for motion artifacts as needed.

*Z* score represents a method of standardization of mapping values which related to the mean normal value of the specific scanner and its standard deviation (SD). The formula for calculating z score is *z* = (*a*–*b*)/*x*, where *a* is the diagnostic T1 cut-off value for CA, b is the mean T1 from the healthy controls, and *x* the SD of the control population [15].

### Statistics

Data analysis is performed using Microsoft Excel and GraphPad Prism. Quantitative data are expressed as mean ± standard deviation or median with 95% confidence intervals, as appropriate. Categorical variables are expressed as numbers and percentages. *p* values were calculated using ANOVA (for continuous variables) with the Tukey test to correct for multiple comparisons or the Chi-square test for categorical variables. Diagnostic accuracy is assessed by receiver operating characteristic curve among patients with increased LV wall thickness.

## Results

### Patient characteristics

One hundred twenty-five consecutive patients were included in the study. 31 subjects without evidence of structural heart disease served as control group. Mean age of patients was 66 ± 14 years, 83% were male. Patients with CA were older, had higher NYHA functional class, higher cardiac biomarkers, more impaired kidney function and suffered frequently from atrial fibrillation compared to the other groups. As expected, patients with CA exhibited frequently extra-cardiac comorbidities such as carpal tunnel syndrome, spinal stenosis and polyneuropathy. Of 21 HCM patients, 12 had typical intramural LGE on inferior or anterior RV insertion points or in the inter-ventricular septum which supported the diagnosis [12]. Family history for HCM was positive in 2 patients, an implantable cardioverter defibrillator was implanted in 2 patients. 4 patients showed LV outflow tract obstruction (i.e. hypertrophic obstructive cardiomyopathy). 3 patients underwent morrow resection or transcoronary ablation of septal hypertrophy (TASH). Patients with HCM differed from those with HHD in terms of age (younger) and in the prevalence of hypertension (less). Patients with HHD had a median of three antihypertensive drugs. There were no patients with severe mitral or aortic valvular lesions or on dialysis in the study. Details of patient characteristics are shown in Table 1.

**Table 1:** Baseline characteristics

	Control (*n* = 31)	Cardiac amyloidosis (*n* = 24)	HCM (*n* = 21)	HHD (*n* = 80)	*p* value
Age, years	53 ± 13	75 ± 8	54 ± 18	66 ± 12	** < 0.0001**
Male sex	19 (61%)	19 (79%)	16 (76%)	69 (86%)	**0.008**
Body mass index	27.9 ± 4.5	26.0 ± 3.3	26.9 ± .44	28.3 ± 4.2	0.12
Systolic BP, mmHg	142 ± 24	132 ± 18	146 ± 21	154 ± 44	0.097
Diastolic BP, mmHg	81 ± 10	76 ± 11	89 ± 21	85 ± 17	0.073
Heart rate, bpm	73 ± 10	78 ± 15	71 ± 15	70 ± 14	0.099
NYHA functional class					
II	0	6 (25%)	9 (43%)	10 (13%)	**0.002**
III	0	15 (63%)	2 (10%)	10 (13%)	** < 0.0001**
IV	0	1 (4%)	0	1 (1%)	0.52
NT-proBNP, pg/mL		3470 (2378–7228)	415 (122–1741)	520 (138–4107)	0.64
Troponin T, pg/mL		54 (43–68)	17 (7–32)	15 (10–36)	0.68
Hemoglobin (mg/dl)	14.7 ± 1.6	13.1 ± 2.0	14.1 ± 1.4	13.2 ± 2.3	0.62
Chronic kidney failure (eGFR < 60 ml/min/1.73m^2^)	0	14 (58%)	2 (10%)	9 (11%)	** < 0.0001**
eGFR, ml/min/1.73m^2^	88 ± 14	58 ± 19	82 ± 22	70 ± 21	** < 0.0001**
Atrial fibrillation	1 (3%)	19 (79%)	5 (24%)	18 (23%)	** < 0.0001**
Coronary artery disease	3 (10%)	12 (50%)	4 (19%)	25 (31%)	**0.007**
Previous MI	1 (3%)	2 (8%)	0	8 (10%)	0.33
Previous PCI or CABG	1 (3%)	5 (21%)	0	14 (18%)	**0.035**
Aortic or mitral disease	0	0	1	4	0.27
Moderate aortic stenosis	0	0	1	2	0.55
Moderate MR	0	0	0	2	0.59
Pacemaker or ICD	0	4 (17%)	3 (14%)	8 (10%)	0.16
Arterial hypertension	5 (16%)	19 (79%)	9 (43%)	80 (100%)	** < 0.0001**
No. of antihypertensives*	2 (2.0–0.02)	3 (2.5–4.5)	2 (1.3–3.8)	3 (2–4)	0.16
Diabetes	0	1 (4%)	0	2 (3%)	0.61
COPD	0	3 (13%)	1 (4%)	4 (5%)	0.22
Cancer	1 (3%)	9 (38%)	1 (4%)	2 (3%)	** < 0.0001**
Multiple myeloma	1 (3%)	6 (25%)	0	0	** < 0.0001**
Other solid or hematologic neoplasms	0	3 (13%)	0	2 (3%)	**0.036**
Carpal tunnel syndrome	0	3 (13%)	0	0	**0.0062**
Spinal stenosis	0	4 (17%)	0	0	**0.0008**
Degenerative joint disease	1 (3%)	5 (21%)	2 (10%)	12 (15%)	0.21
Non-traumatic tendon rupture	0	0	0	1 (1%)	1.0
Polyneuropathy	0	7 (29%)	0	0	** < 0.0001**

Bold values indicate statistically significant *p* values with *p*< 0.05

Values are mean ± standard deviation, median (interquartile range) or numbers (percent). * among patients with antihypertensive medical therapy

*ACB* aorto-coronary bypass; *BP* blood pressure; *COPD* chronic obstructive pulmonary disease; *eGFR* estimated glomerular filtration rate using CKD EPI formula; *ICD* implantable cardioverter defibrillator; *MI* myocardial infarction; *MR* mitral regurgitation; *PCI* percutaneous coronary intervention

### Myocardial remodeling and morphology

**Fig. 2:**
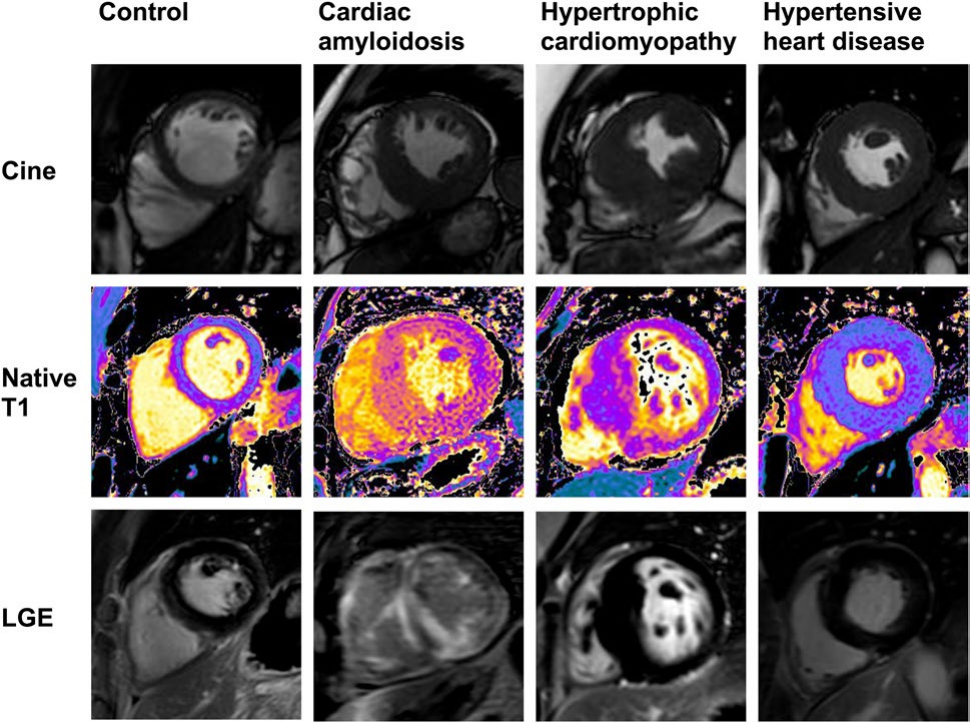
CMR tissue characterization in hypertrophic phenotypes. Cardiovascular magnetic resonance (CMR) end-diastolic left ventricular short axis cine images (top raw), modified Look-Locker inversion recovery native T1 mapping (middle raw) and late gadolinium enhancement (LGE) by phase-sensitive inverse recovery (bottom raw) in healthy control, patients with cardiac amyloidosis, hypertrophic cardiomyopathy and hypertensive heart disease

**Fig. 3:**
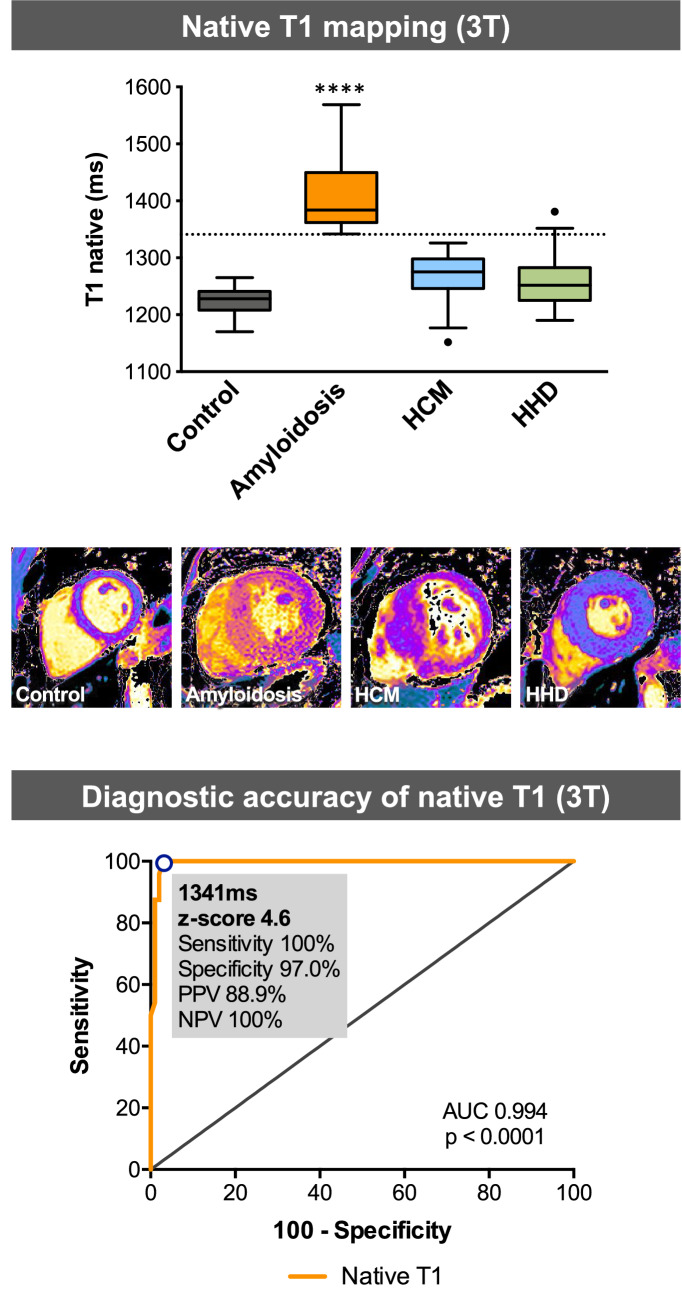
Summary figure. Top, native T1 mapping values and representative short axis T1 images of controls, patients with cardiac amyloidosis (CA), hypertrophic cardiomyopathy (HCM) and hypertensive heart disease (HHD). The dotted line represents the optimal cut-off value (1341 ms) for native T1 mapping to differentiate CA from HCM and HHD. Bottom, ROC curve of native T1 revealed 1341 ms as the best cut-off values for the diagnosis of CA vs. patients with HCM and HHD. *AUC* area under the curve; *PPV* positive predicted value; *NPV* negative predicted values. ****p < 0.0001 vs. Control, HCM and HHD

Both patients with CA and HCM had increased LV wall thickness and LV mass index compared to controls and HHD (*p* < 0.0001), indicating advanced concentric LV remodeling (Table 2). Consecutively, LV end-diastolic diameter, stroke volume and ejection fraction (EF) were lower in patients with CA (*p* < 0.0001). All patients with CA and 81% of patients with HCM showed LGE. Quantitative LGE was highest in patients with CA (55 ± 32%) compared to HCM (10 ± 9%) and HHD (3 ± 6%) (*p* < 0.0001). Native T1 times were higher in patients with CA (1409 ± 59 ms) compared to all other groups (*p* < 0.0001; Figs. 2 and 3). Regional differences of T1 values were moderate; the mean values of segmental standard deviations were 36 ms for controls, 82 ms for amyloidosis, 56 ms for HCM, and 61 ms for HHD (full T1 data of myocardial segments are available in the supplement tables). ECV was highest in patients with CA (51 ± 6%) compared to HCM (30 ± 6%) and HHD (31 ± 6%) (*p* < 0.0001). Detailed CMR data are shown in Table 2.

**Table 2:** CMR data

	Control (*n* = 31)	Cardiac amyloidosis (*n* = 24)	HCM (*n* = 21)	HHD (*n* = 80)	*p* value
IVSd, mm	10 ± 1	18 ± 4^*‡^	17 ± 4^*‡^	14 ± 2^*†^	** < 0.0001**
LVEDD, mm	50 ± 4	47 ± 5^‡^	47 ± 6^‡^	50 ± 5^†^	**0.0016**
LVEDV index, mL/m^2^	55 ± 8	50 ± 11	51 ± 10	54 ± 15	0.42
LVESV index, mL/m^2^	18 ± 5	21 ± 8	15 ± 7	19 ± 9	0.15
SV index, mL/m^2^	36 ± 5	29 ± 8^*†‡^	37 ± 9	35 ± 8	**0.0026**
EF, %	67 ± 6	58 ± 11^*†‡^	71 ± 11	66 ± 9	** < 0.0001**
LV mass index, g/m^2^	52 ± 11	87 ± 22^*‡^	87 ± 29^*^	73 ± 17^*^	** < 0.0001**
LGE present	0	24 (100%)	17 (81%)	29 (36%)	** < 0.0001**
LGE quantitative, %	0	55 ± 32^*†‡^	10 ± 9	3 ± 6	** < 0.0001**
Native T1, ms	1225 ± 21	1409 ± 59^*†‡^	1266 ± 44^*^	1257 ± 41^*^	** < 0.0001**
ECV, %	26 ± 3	51 ± 6^*†‡^	30 ± 6	31 ± 6^*^	** < 0.0001**

Bold values indicate statistically significant *p* values with *p*< 0.05

Values are mean ± standard deviation or numbers (%). ECV data available for *n* = 12 (control), *n* = 22 (cardiac amyloidosis), *n* = 14 (HCM), *n* = 32 (HDD)

*HCM* hypertrophic cardiomyopathy; *HHD* hypertensive heart disease; *IVSD* inter-ventricular septum thickness at end-diastole; *EF* LV ejection fraction; *ECV* extracellular volume; *LGE* late gadolinium enhancement; *LV* left ventricle; *LVEDD* LV end-diastolic dimeter; *LVEDV* LV end-diastolic volume; *LVESV* LV end-diastolic volume; *SV* stroke volume

*p < 0.05 vs. control

^†^*p* < 0.05 vs. HCM

^‡^*p* < 0.05 vs. HHD

### ROC curves of CMR parameters

**Fig. 4:**
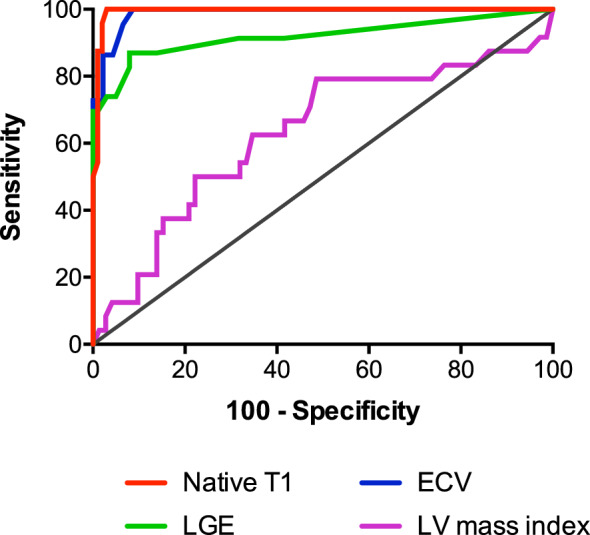
Diagnostic accuracy of CMR parameters for the diagnosis of cardiac amyloidosis. Receiver-operating characteristic (ROC) curves of CMR parameters for the diagnosis of cardiac amyloidosis vs. hypertrophic cardiomyopathy (HCM) and hypertensive heart disease (HHD). *ECV* extracellular volume; *LGE* late gadolinium enhancement

Diagnostic accuracy of native T1, ECV, quantitative LGE and LV mass index to diagnose CA among patients with increased LV wall thickness was assessed on ROC curve. Native T1 showed a higher area under the curve (AUC) of 0.9938 (*p* < 0.0001; Table 3, Fig. 4) compared to ECV (0.9876, *p* < 0.0001), quantitative LGE (0.9406 *p* < 0.0001) and LV mass index (0.6296, *p* = 0.058). The best cut-off value of native T1 mapping to diagnose CA was 1341 ms with a *z* score of 4.6. For this cut-off value, sensitivity was 100% and specificity 97.0%. The positive and negative predicted values were 88.9% and 100%, respectively (Fig. 3, bottom).

**Table 3:** Diagnostic performance of CMR parameters for the diagnosis of cardiac amyloidosis

	AUC (95% CI)	*p* value
Native T1 mapping	0.9938 (0.984–1.004)	**< 0.0001**
LGE quantitative	0.9406 (0.884–0.997)	** < 0.0001**
ECV	0.9876 (0.970–1.001)	**< 0.0001**
LV mass index	0.6296 (0.491–0.768)	0.058

Bold values indicate statistically significant *p* values with *p*< 0.05

*ATTR* transthyretin amyloidosis; *AUC* area under the curve; *CI* confidence interval; *CMR* cardiac magnetic resonance tomography; *ECV* extracellular volume; *LGE* late gadolinium enhancement; *LV* left ventricle

## Discussion

The study shows that cardiac amyloidosis is characterized by increased native T1 times of the LV myocardium compared to healthy myocardium, HCM and HHD. Native T1 mapping has high diagnostic accuracy to detect CA among patients with increased LV wall thickness. Therefore, CMR with native T1 mapping provides a diagnostic tool to diagnose CA in patients with LV hypertrophy without contrast medium.

### Native T1 in cardiac amyloidosis

Morphological and functional evaluation is of particular importance in patients with LV hypertrophy. The European Society of Cardiology guidelines recommend CMR in patients with suspected HCM or CA [6, 16]. Patients with CA show distinct CMR characteristics, i.e. increased LV mass, lower LVEF and diffuse LGE. However, these alterations are not specific for CA. A recent German study compared diagnostic accuracy of CMR with endomyocardial biopsy (EMB) as reference method in 160 patients [17]. The authors showed that a specific pattern of LGE suggestive of CA had a high diagnostic accuracy for the diagnosis of CA. However, this retrospective study was restricted to patients who had undergone both CMR and EMB. Therefore, a selection bias cannot be excluded. The results might be affected by the presence, the amount or the pattern of LGE, which might explain the lower diagnostic accuracy of native T1 and ECV in this population.

Native T1 mapping is a sensitive method to detect myocardial interstitial remodeling and adds incremental information for characterization of hypertrophic cardiomyopathies [6, 9, 18]. Native T1 mapping allows differentiation of patients with HCM from HHD [19]. Baggiano et al. studied a large patient population with suspected CA who were referred to the UK national amyloidosis center [11], which suggests a relevant pre-selection. Based on their data, the authors propose a non-contrast CMR approach with native T1 specific cut-off values (1.5 T) for both exclusion and definite diagnosis of CA. The administration of contrast agent for LGE and ECV calculation would be restricted to non-diagnostic T1 times. Another analysis from the same group showed similar accuracy of T1 and ECV in terms of the diagnosis of CA compared to HCM and asymptomatic transthyretin gene mutation carriers [20]. This was further confirmed in a meta-analysis, which demonstrated similar diagnostic performance of native T1 compared to both LGE and ECV for the diagnosis of CA [21]. Among these parameters, native T1 is the only method that does not require contrast agent. Therefore, this technique is promising in the diagnosis and screening in patients at risk for CA.

Of note, a hypertrophic phenotype was not an inclusion criterium in the studies mentioned above. This is an important difference to our study. Our population consisted of consecutive patients with increased LV wall thickness without pre-selection representing a broad real-world population. In this cohort, native T1 mapping identified patients with CA with high diagnostic accuracy that was superior to LGE and ECV. The lower diagnostic accuracy of ECV compared to other reports [17, 21] might be due differences in the patient population and limited availability of ECV values. With an identified optimal native T1 cut-off value of 1341 ms, all patients with CA would be diagnosed correctly, while 3 patients would be falsely positive. In patients with increased LV wall thickness, CA is an important differential diagnosis with therapeutic consequences. This is particularly important because CA is underdiagnosed due to the lack of specific signs and symptoms. There is a high need for specific diagnostic criteria of CA in cardiac imaging such as CMR, as well as a simple method for screening of patients at risk for CA. Until now, there have been no specific cut-off values for the diagnosis of CA of native T1 for the diagnosis of CA in 3 T CMR scanner available, which provide higher special resolution compared to 1.5 T [22].

Our data provide several new findings that are of practical importance for the diagnosis of CA by CMR: (A) Native T1 mapping in patients with increased LV wall thickness who underwent CMR identifies CA with a very high diagnostic accuracy. (B) We identified a cutoff of 1341 ms native T1 time in the 3 T scanner for the diagnosis of CA with a sensitivity of 98% and a sensitivity of 100%. (C) Non-contrast CMR represents a useful tool for a simple imaging approach to patients with increased LV wall thickness who are at risk for CA.

### Diagnostic approach to CA

The diagnosis of CA is currently established by bone scintigraphy using ^99^Tc-DPD in combination with blood test for monoclonal protein or endomyocardial biopsy [1, 4]. Scintigraphy is based on radioactive tracers, and it does not allow differential diagnosis in case of no myocardial tracer uptake. Endomyocardial biopsy is an invasive procedure with inherent risks. CMR provides diagnostic information about the dimension, function and structure of the heart and facilitates differential diagnosis. Native myocardial T1 imaging does not require the administration of contrast agent [10, 23, 24]. This is particularly important in CA because amyloidosis frequently affects the kidneys precluding contrast administration when renal function is severely impaired. Current data suggest that native T1 times correspond to transthyretin amyloid burden in the myocardium, and may be elevated even at an early stage of the disease [25]. Therefore, native T1 mapping provides a rapid, non-invasive, non-contrast and non-radiation method for evaluation of patients at risk for CA. For this purpose, CMR with native T1 mapping might be an optimal imaging method in elderly patients with increased LV wall thickness. Our study provides the basis for an ongoing prospective clinical trial to evaluate the diagnostic role of CMR for the detection of CA in symptomatic patients with increased LV wall thickness (NCT04862273).

### Comparing native T1 values

Many physical factors influence T1 values, such as magnetic field strength [26]. Thus, comparing the absolute T1 values of 1.5 T and 3 T scanners is not reliable [15, 22]. 3 T CMR scanners provide higher spatial resolution compared to 1.5 T, but reference T1 values to diagnose CA are currently lacking [22]. Our data provide important cut-off values for the diagnosis of CA for 3 T CMR scanners.

*Z* score mapping has been developed to compare mapping values between different CMR vendors and magnetic field strengths. A recent study demonstrated a similar diagnostic accuracy of *z*-score mapping compared to native T1 to diagnose CA [15]. Our ROC analysis revealed a *z* score of 4.6 (corresponding to 1341 ms) for the diagnosis of CA. The higher *z*-score value in our study compared to others might be attributed to the comparison of patients with other cardiac pathologies, i.e. HCM and HHD, instead of healthy volunteers [15] or a heterogeneous population including a high number of normal hearts [11]. Thus, the patient population is of great importance when z score will be transferred to a clinical application, and a prospective validation of z-score mapping is needed.

### Limitations

This is a monocentric, retrospective study with its inherent limitations. The overall number of 125 patients in the study is a limitation and prohibits further subgroup analyses. The clinical data, derived from the university hospital data warehouse, may be incomplete due to the retrospective nature of the study. Details of potential genetic testing of HCM patients were not available for this study. Hematocrit values for ECV calculation were available in a limited number of patients only because diagnostic workup was performed in the outpatient setting in many patients. Most patients with CA in our study are in an advanced disease stage, indicated by pronounced LV remodeling and LGE. We included consecutive patients with a hypertrophic phenotype who underwent clinical CMR, but a referral bias of outpatients might be present. Therefore, the data require confirmation in patients at early stages of CA.

### Conclusion

Patients with cardiac amyloidosis showed elevated native T1 times of the LV myocardium compared to those with hypertrophic cardiomyopathy, hypertensive heart disease and healthy control. Native T1 mapping exhibits a high diagnostic accuracy for the diagnosis of CA among patients with increased LV wall thickness. Thus, the study identifies native T1 mapping as a useful method for the diagnosis of CA in patients with LV hypertrophy. The new T1 cut-off data on 3 T CMR provide the basis for prospective clinical trial to evaluate non-contrast CMR with native T1 mapping in patients who are at high risk for cardiac amyloidosis.

## Supplementary Information

Below is the link to the electronic supplementary material.

Supplementary file1 (DOCX 65 KB)
