# Supplementary material for: Native T1 mapping for the diagnosis of cardiac amyloidosis in patients with left ventricular hypertrophy
Source: Clin Res Cardiol. 2022 Mar 31;112(3):334–42. doi: 10.1007/s00392-022-02005-2 (PMC9998594; doi:10.1007/s00392-022-02005-2)
Supplement: Supplementary file 1 — Supplementary file1 (DOCX 65 KB) [file 392_2022_2005_MOESM1_ESM.docx]

**Supplement Tables**

Native T1 values in 18 myocardial segments of the study participants, according to group.

**Control**

| **No.** | **basal** | | | | | | **mid-ventricular** | | | | | | **apical** | | | | | |
| --- | --- | --- | --- | --- | --- | --- | --- | --- | --- | --- | --- | --- | --- | --- | --- | --- | --- | --- |
| 1 | 1225 | 1294 | 1280 | 1247 | 1234 | 1213 | 1206 | 1275 | 1245 | 1250 | 1237 | 1223 | 1194 | 1227 | 1234 | 1214 | 1243 | 1225 |
| 2 | 1114 | 1210 | 1183 | 1269 | 1233 | 1181 | 1153 | 1188 | 1196 | 1198 | 1236 | 1182 | 1193 | 1210 | 1266 | 1277 | 1193 | 1182 |
| 3 | 1274 | 1220 | 1256 | 1298 | 1252 | 1253 | 1273 | 1255 | 1359 | 1216 | 1195 | 1226 | 1240 | 1221 | 1297 | 1229 | 1292 | 1294 |
| 4 | 1163 | 1227 | 1216 | 1258 | 1222 | 1171 | 1144 | 1205 | 1213 | 1223 | 1200 | 1176 | 1204 | 1179 | 1212 | 1237 | 1224 | 1253 |
| 5 | 1157 | 1255 | 1204 | 1188 | 1169 | 1212 | 1205 | 1220 | 1226 | 1177 | 1160 | 1233 | 1277 | 1208 | 1220 | 1205 | 1209 | 1257 |
| 6 | 1152 | 1220 | 1197 | 1235 | 1229 | 1130 | 1151 | 1212 | 1173 | 1180 | 1230 | 1203 | 1163 | 1236 | 1230 | 1220 | 1220 | 1178 |
| 7 | 1209 | 1226 | 1223 | 1237 | 1197 | 1168 | 1268 | 1280 | 1257 | 1238 | 1182 | 1181 | 1287 | 1280 | 1291 | 1266 | 1253 | 1315 |
| 8 | 1155 | 1258 | 1192 | 1211 | 1217 | 1165 | 1135 | 1208 | 1207 | 1192 | 1184 | 1182 | 1182 | 1201 | 1194 | 1163 | 1191 | 1184 |
| 9 | 1131 | 1201 | 1198 | 1207 | 1172 | 1144 | 1160 | 1192 | 1197 | 1231 | 1124 | 1167 | 1094 | 1186 | 1199 | 1105 | 1144 | 1183 |
| 10 | 1255 | 1267 | 1240 | 1197 | 1167 | 1177 | 1200 | 1225 | 1222 | 1228 | 1209 | 1185 | 1226 | 1195 | 1188 | 1207 | 1188 | 1202 |
| 11 | 1231 | 1246 | 1258 | 1201 | 1226 | 1200 | 1215 | 1203 | 1231 | 1237 | 1175 | 1241 | 1247 | 1280 | 1241 | 1230 | 1248 | 1236 |
| 12 | 1204 | 1235 | 1230 | 1264 | 1199 | 1183 | 1214 | 1214 | 1236 | 1171 | 1335 | 1219 | 1201 | 1209 | 1237 | 1226 | 1288 | 1216 |
| 13 | 1193 | 1295 | 1242 | 1255 | 1243 | 1227 | 1208 | 1240 | 1258 | 1290 | 1188 | 1183 | 1240 | 1269 | 1287 | 1257 | 1293 | 1293 |
| 14 | 1203 | 1257 | 1208 | 1259 | 1204 | 1180 | 1229 | 1241 | 1241 | 1259 | 1249 | 1206 | 1247 | 1262 | 1239 | 1246 | 1254 | 1269 |
| 15 | 1206 | 1230 | 1256 | 1215 | 1177 | 1168 | 1203 | 1261 | 1221 | 1211 | 1202 | 1127 | 1161 | 1215 | 1259 | 1206 | 1232 | 1201 |
| 16 | 1161 | 1269 | 1233 | 1335 | 1196 | 1201 | 1225 | 1262 | 1231 | 1152 | 1313 | 1235 | 1250 | 1272 | 1258 | 1188 | 1231 | 1255 |
| 17 | 1242 | 1270 | 1261 | 1244 | 1171 | 1185 | 1199 | 1207 | 1221 | 1205 | 1200 | 1193 | 1145 | 1207 | 1214 | 1188 | 1205 | 1223 |
| 18 | 1194 | 1259 | 1183 | 1200 | 1250 | 1169 | 1227 | 1210 | 1253 | 1212 | 1211 | 1201 | 1264 | 1251 | 1230 | 1283 | 1240 | 1234 |
| 19 | 1219 | 1233 | 1212 | 1229 | 1198 | 1181 | 1239 | 1234 | 1221 | 1232 | 1213 | 1231 | 1324 | 1287 | 1273 | 1248 | 1257 | 1390 |
| 20 | 1127 | 1240 | 1222 | 1231 | 1189 | 1150 | 1114 | 1208 | 1225 | 1228 | 1204 | 1175 | 1139 | 1253 | 1238 | 1217 | 1209 | 1167 |
| 21 | 1275 | 1270 | 1273 | 1233 | 1203 | 1224 | 1257 | 1261 | 1261 | 1215 | 1191 | 1214 | 1264 | 1276 | 1266 | 1225 | 1247 | 1251 |
| 22 | 1232 | 1252 | 1221 | 1254 | 1219 | 1206 | 1195 | 1243 | 1237 | 1300 | 1241 | 1258 | 1172 | 1224 | 1280 | 1266 | 1191 | 1187 |
| 23 | 1238 | 1277 | 1219 | 1229 | 1197 | 1192 | 1217 | 1213 | 1235 | 1242 | 1174 | 1182 | 1274 | 1250 | 1229 | 1252 | 1265 | 1243 |
| 24 | 1245 | 1268 | 1252 | 1269 | 1287 | 1179 | 1221 | 1229 | 1235 | 1257 | 1256 | 1181 | 1267 | 1315 | 1260 | 1247 | 1257 | 1255 |
| 25 | 1203 | 1276 | 1243 | 1244 | 1214 | 1209 | 1222 | 1288 | 1255 | 1247 | 1255 | 1228 | 1373 | 1327 | 1277 | 1298 | 1311 | 1269 |
| 26 | 1252 | 1226 | 1209 | 1261 | 1184 | 1184 | 1188 | 1191 | 1243 | 1332 | 1324 | 1150 | 1192 | 1171 | 1240 | 1213 | 1191 | 1226 |
| 27 | 1220 | 1229 | 1256 | 1255 | 1205 | 1216 | 1223 | 1256 | 1214 | 1269 | 1238 | 1207 | 1241 | 1198 | 1225 | 1254 | 1249 | 1268 |
| 28 | 1204 | 1243 | 1204 | 1215 | 1167 | 1150 | 1213 | 1199 | 1218 | 1191 | 1144 | 1226 | 1228 | 1203 | 1184 | 1243 | 1189 | 1247 |
| 29 | 1163 | 1234 | 1269 | 1243 | 1207 | 1289 | 1235 | 1235 | 1248 | 1273 | 1186 | 1217 | 1261 | 1254 | 1281 | 1238 | 1246 | 1240 |
| 30 | 1228 | 1227 | 1232 | 1208 | 1200 | 1214 | 1206 | 1216 | 1234 | 1225 | 1219 | 1208 | 1223 | 1219 | 1211 | 1209 | 1176 | 1194 |
| 31 | 1191 | 1239 | 1240 | 1232 | 1207 | 1206 | 1196 | 1242 | 1286 | 1260 | 1207 | 1172 | 1234 | 1296 | 1318 | 1285 | 1236 | 1260 |

**Cardiac amyloidosis**

| **No.** | **basal** | | | | | | **mid-ventricular** | | | | | | **apical** | | | | | |
| --- | --- | --- | --- | --- | --- | --- | --- | --- | --- | --- | --- | --- | --- | --- | --- | --- | --- | --- |
| 1 | 1253 | 1331 | 1472 | 1380 | 1292 | 1266 | 1262 | 1288 | 1388 | 1366 | 1275 | 1231 | 1321 | 1345 | 1398 | 1485 | 1421 | 1385 |
| 2 | 1375 | 1434 | 1395 | 1486 | 1530 | 1336 | 1346 | 1377 | 1420 | 1394 | 1442 | 1320 | 1569 | 1443 | 1444 | 1371 | 1398 | 1453 |
| 3 | 1302 | 1305 | 1309 | 1448 | 1340 | 1318 | 1368 | 1369 | 1253 | 1318 | 1412 | 1340 | 1450 | 1428 | 1360 | 1253 | 1297 | 1346 |
| 4 | 1458 | 1508 | 1506 | 1489 | 1510 | 1446 | 1485 | 1572 | 1567 | 1564 | 1558 | 1496 | 1479 | 1524 | 1595 | 1503 | 1536 | 1474 |
| 5 | 1326 | 1348 | 1349 | 1467 | 1321 | 1327 | 1325 | 1254 | 1289 | 1256 | 1381 | 1356 | 1304 | 1293 | 1380 | 1400 | 1461 | 1373 |
| 6 | 1284 | 1348 | 1321 | 1384 | 1325 | 1342 | 1319 | 1406 | 1405 | 1324 | 1340 | 1336 | 1415 | 1420 | 1405 | 1365 | 1368 | 1454 |
| 7 | 1362 | 1375 | 1380 | 1391 | 1336 | 1344 | 1373 | 1417 | 1439 | 1340 | 1348 | 1362 | 1338 | 1357 | 1398 | 1353 | 1318 | 1348 |
| 8 | 1421 | 1442 | 1433 | 1469 | 1457 | 1373 | 1435 | 1488 | 1509 | 1493 | 1430 | 1404 | 1445 | 1489 | 1537 | 1482 | 1446 | 1435 |
| 9 | 1495 | 1480 | 1520 | 1473 | 1435 | 1375 | 1430 | 1483 | 1504 | 1497 | 1469 | 1410 | 1459 | 1472 | 1456 | 1442 | 1465 | 1414 |
| 10 | 1372 | 1394 | 1399 | 1426 | 1376 | 1396 | 1341 | 1403 | 1403 | 1342 | 1273 | 1305 | 1332 | 1397 | 1453 | 1382 | 1326 | 1318 |
| 11 | 1390 | 1427 | 1404 | 1495 | 1434 | 1376 | 1522 | 1422 | 1437 | 1468 | 1468 | 1464 | 1392 | 1409 | 1424 | 1446 | 1453 | 1373 |
| 12 | 1299 | 1348 | 1343 | 1407 | 1377 | 1274 | 1307 | 1382 | 1404 | 1361 | 1316 | 1255 | 1479 | 1389 | 1383 | 1381 | 1431 | 1436 |
| 13 | 1329 | 1342 | 1373 | 1439 | 1372 | 1247 | 1237 | 1370 | 1398 | 1398 | 1303 | 1270 | 1259 | 1382 | 1411 | 1423 | 1317 | 1275 |
| 14 | 1283 | 1345 | 1320 | 1383 | 1320 | 1340 | 1326 | 1406 | 1409 | 1323 | 1340 | 1337 | 1411 | 1424 | 1405 | 1366 | 1369 | 1453 |
| 15 | 1321 | 1427 | 1421 | 1461 | 1488 | 1379 | 1532 | 1610 | 1426 | 1428 | 1444 | 1412 | 1570 | 1427 | 1499 | 1397 | 1517 | 1381 |
| 16 | 1295 | 1363 | 1371 | 1401 | 1401 | 1345 | 1464 | 1401 | 1495 | 1380 | 1338 | 1398 | 1406 | 1344 | 1296 | 1351 | 1358 | 1382 |
| 17 | 1265 | 1421 | 1394 | 1348 | 1328 | 1323 | 1314 | 1419 | 1410 | 1368 | 1333 | 1340 | 1300 | 1377 | 1405 | 1370 | 1345 | 1302 |
| 18 | 1367 | 1407 | 1399 | 1444 | 1445 | 1379 | 1415 | 1468 | 1487 | 1483 | 1495 | 1446 | 1483 | 1458 | 1507 | 1513 | 1484 | 1522 |
| 19 | 1250 | 1433 | 2453 | 1392 | 1422 | 1164 | 1352 | 1457 | 1439 | 1363 | 1322 | 1145 | 1373 | 1445 | 1487 | 1276 | 1407 | 1395 |
| 20 | 1362 | 1460 | 1501 | 1455 | 1456 | 1380 | 1347 | 1519 | 1543 | 1461 | 1453 | 1368 | 1333 | 1487 | 1542 | 1469 | 1514 | 1348 |
| 21 | 1614 | 1526 | 1556 | 1589 | 1679 | 1642 | 1556 | 1519 | 1576 | 1589 | 1606 | 1530 | 1461 | 1528 | 1584 | 1606 | 1498 | 1484 |
| 22 | 1432 | 1471 | 1473 | 1546 | 1563 | 1369 | 1338 | 1402 | 1513 | 1482 | 1435 | 1322 | 1500 | 1460 | 1464 | 1446 | 1415 | 1496 |
| 23 | 1378 | 1456 | 1456 | 1414 | 1396 | 1315 | 1350 | 1439 | 1445 | 1390 | 1348 | 1323 | 1363 | 1414 | 1443 | 1380 | 1345 | 1297 |
| 24 | 1399 | 1424 | 1463 | 1466 | 1400 | 1379 | 1399 | 1454 | 1483 | 1427 | 1383 | 1401 | 1387 | 1454 | 1469 | 1435 | 1392 | 1378 |

**Hypertrophic cardiomyopathy**

| **No.** | **basal** | | | | | | **mid-ventricular** | | | | | | **apical** | | | | | |
| --- | --- | --- | --- | --- | --- | --- | --- | --- | --- | --- | --- | --- | --- | --- | --- | --- | --- | --- |
| 1 | 1266 | 1300 | 1381 | 1472 | 1245 | 1248 | 1167 | 1249 | 1348 | 1349 | 1262 | 1242 | 1188 | 1318 | 1430 | 1394 | 1110 | 1297 |
| 2 | 1336 | 1349 | 1388 | 1250 | 1353 | 1308 | 1332 | 1369 | 1315 | 1353 | 1352 | 1384 | 1249 | 1284 | 1371 | 1260 | 1324 | 1216 |
| 3 | 1173 | 1257 | 1326 | 1269 | 1232 | 1257 | 1242 | 1301 | 1326 | 1307 | 1254 | 1236 | 1257 | 1282 | 1342 | 1335 | 1271 | 1263 |
| 4 | 1231 | 1301 | 1291 | 1326 | 1302 | 1216 | 1270 | 1338 | 1312 | 1307 | 1265 | 1246 | 1294 | 1280 | 1333 | 1315 | 1287 | 1307 |
| 5 | 1190 | 1304 | 1269 | 1243 | 1209 | 1305 | 1224 | 1257 | 1246 | 1238 | 1250 | 1242 | 1253 | 1201 | 1273 | 1276 | 1259 | 1264 |
| 6 | 1166 | 1197 | 1196 | 1202 | 1155 | 1170 | 1163 | 1156 | 1160 | 1198 | 1200 | 1199 | 1182 | 1168 | 1176 | 1149 | 1194 | 1164 |
| 7 | 1202 | 1259 | 1258 | 1272 | 1291 | 1224 | 1213 | 1225 | 1225 | 1261 | 1213 | 1208 | 1184 | 1235 | 1233 | 1101 | 1180 | 1215 |
| 8 | 1164 | 1262 | 1234 | 1165 | 1154 | 1170 | 1143 | 1126 | 1140 | 1165 | 1183 | 1199 | 1178 | 1168 | 1131 | 1128 | 1149 | 1138 |
| 9 | 1251 | 1298 | 1271 | 1324 | 1302 | 1236 | 1269 | 1318 | 1302 | 1247 | 1220 | 1247 | 1324 | 1265 | 1354 | 1315 | 1286 | 1305 |
| 10 | 1356 | 1245 | 1271 | 1260 | 1307 | 1315 | 1273 | 1269 | 1282 | 1241 | 1155 | 1264 | 1229 | 1269 | 1294 | 1475 | 1276 | 1266 |
| 11 | 1291 | 1329 | 1280 | 1294 | 1284 | 1206 | 1309 | 1339 | 1377 | 1324 | 1269 | 1255 | 1358 | 1324 | 1318 | 1339 | 1337 | 1306 |
| 12 | 1278 | 1258 | 1279 | 1241 | 1238 | 1250 | 1297 | 1287 | 1261 | 1230 | 1308 | 1258 | 1273 | 1269 | 1296 | 1224 | 1278 | 1289 |
| 13 | 1223 | 1291 | 1309 | 1287 | 1235 | 1223 | 1222 | 1288 | 1292 | 1427 | 1291 | 1284 | 1274 | 1253 | 1295 | 1314 | 1280 | 1311 |
| 14 | 1234 | 1283 | 1294 | 1280 | 1252 | 1203 | 1260 | 1282 | 1313 | 1293 | 1235 | 1243 | 1262 | 1320 | 1326 | 1290 | 1217 | 1217 |
| 15 | 1273 | 1249 | 1243 | 1254 | 1212 | 1238 | 1246 | 1218 | 1215 | 1219 | 1218 | 1200 | 1242 | 1337 | 1390 | 1229 | 1240 | 1208 |
| 16 | 1286 | 1283 | 1366 | 1272 | 1267 | 1269 | 1293 | 1296 | 1291 | 1286 | 1262 | 1279 | 1262 | 1297 | 1299 | 1279 | 1259 | 1258 |
| 17 | 1340 | 1384 | 1379 | 1313 | 1238 | 1351 | 1310 | 1304 | 1336 | 1323 | 1191 | 1291 | 1260 | 1272 | 1261 | 1334 | 1267 | 1275 |
| 18 | 1292 | 1330 | 1284 | 1292 | 1284 | 1229 | 1310 | 1341 | 1370 | 1344 | 1270 | 1254 | 1359 | 1324 | 1308 | 1339 | 1338 | 1306 |
| 19 | 1181 | 1291 | 1309 | 1287 | 1235 | 1167 | 1222 | 1288 | 1292 | 1376 | 1291 | 1234 | 1274 | 1253 | 1300 | 1314 | 1280 | 1311 |
| 20 | 1225 | 1263 | 1251 | 1269 | 1188 | 1251 | 1276 | 1247 | 1243 | 1137 | 1191 | 1208 | 1206 | 1203 | 1235 | 1039 | 1094 | 1212 |
| 21 | 1274 | 1312 | 1283 | 1253 | 1265 | 1251 | 1267 | 1228 | 1267 | 1244 | 1243 | 1245 | 1227 | 1226 | 1213 | 1231 | 1238 | 1263 |

**Hypertensive heart disease**

| **No.** | **basal** | | | | | | **mid-ventricular** | | | | | | **apical** | | | | | |
| --- | --- | --- | --- | --- | --- | --- | --- | --- | --- | --- | --- | --- | --- | --- | --- | --- | --- | --- |
| 1 | 1235 | 1318 | 1292 | 1262 | 1314 | 1236 | 1257 | 1262 | 1272 | 1260 | 1256 | 1278 | 1216 | 1244 | 1306 | 1378 | 1269 | 1169 |
| 2 | 1228 | 1222 | 1240 | 1334 | 1281 | 1236 | 1224 | 1224 | 1238 | 1293 | 1278 | 1249 | 1207 | 1194 | 1225 | 1285 | 1296 | 1239 |
| 3 | 1151 | 1235 | 1245 | 1250 | 1237 | 1204 | 1166 | 1224 | 1232 | 1255 | 1272 | 1154 | 1247 | 1226 | 1246 | 1259 | 1235 | 1266 |
| 4 | 1214 | 1274 | 1223 | 1295 | 1377 | 1293 | 1277 | 1267 | 1283 | 1287 | 1334 | 1318 | 1259 | 1278 | 1257 | 1351 | 1360 | 1226 |
| 5 | 1244 | 1253 | 1334 | 1320 | 1194 | 1224 | 1248 | 1264 | 1263 | 1197 | 1225 | 1300 | 1295 | 1218 | 1280 | 1340 | 1270 | 1282 |
| 6 | 1273 | 1263 | 1311 | 1265 | 1255 | 1245 | 1267 | 1222 | 1267 | 1326 | 1234 | 1261 | 1230 | 1221 | 1275 | 1257 | 1260 | 1200 |
| 7 | 1365 | 1444 | 1460 | 1496 | 1405 | 1318 | 1367 | 1404 | 1454 | 1494 | 1410 | 1347 | 1304 | 1271 | 1294 | 1324 | 1325 | 1336 |
| 8 | 1220 | 1240 | 1240 | 1251 | 1221 | 1187 | 1256 | 1260 | 1291 | 1264 | 1195 | 1248 | 1209 | 1258 | 1269 | 1236 | 1270 | 1257 |
| 9 | 1205 | 1213 | 1222 | 1211 | 1218 | 1173 | 1191 | 1237 | 1210 | 1201 | 1214 | 1239 | 1185 | 1180 | 1201 | 1215 | 1207 | 1181 |
| 10 | 1142 | 1218 | 1219 | 1249 | 1247 | 1188 | 1180 | 1230 | 1231 | 1258 | 1191 | 1206 | 1197 | 1210 | 1210 | 1164 | 1160 | 1230 |
| 11 | 1252 | 1270 | 1301 | 1323 | 1367 | 1326 | 1237 | 1274 | 1299 | 1293 | 1297 | 1307 | 1204 | 1273 | 1324 | 1291 | 1292 | 1291 |
| 12 | 1229 | 1221 | 1222 | 1211 | 1212 | 1235 | 1213 | 1254 | 1250 | 1258 | 1260 | 1208 | 1256 | 1262 | 1211 | 1302 | 1236 | 1259 |
| 13 | 1215 | 1273 | 1222 | 1283 | 1377 | 1293 | 1276 | 1265 | 1279 | 1287 | 1324 | 1320 | 1256 | 1277 | 1257 | 1340 | 1345 | 1226 |
| 14 | 1287 | 1306 | 1278 | 1309 | 1356 | 1202 | 1239 | 1264 | 1258 | 1252 | 1257 | 1252 | 1192 | 1255 | 1243 | 1212 | 1199 | 1212 |
| 15 | 1279 | 1307 | 1280 | 1314 | 1359 | 1215 | 1248 | 1268 | 1252 | 1249 | 1208 | 1246 | 1182 | 1200 | 1255 | 1217 | 1199 | 1220 |
| 16 | 1189 | 1238 | 1232 | 1218 | 1179 | 1187 | 1190 | 1228 | 1207 | 1235 | 1220 | 1222 | 1185 | 1213 | 1230 | 1200 | 1225 | 1211 |
| 17 | 1220 | 1206 | 1204 | 1245 | 1305 | 1203 | 1195 | 1223 | 1222 | 1202 | 1225 | 1207 | 1264 | 1234 | 1237 | 1213 | 1209 | 1212 |
| 18 | 1265 | 1247 | 1181 | 1224 | 1276 | 1192 | 1228 | 1229 | 1145 | 1207 | 1216 | 1257 | 1361 | 1276 | 1248 | 1145 | 1267 | 1325 |
| 19 | 1252 | 1300 | 1333 | 1312 | 1279 | 1238 | 1298 | 1326 | 1391 | 1373 | 1310 | 1324 | 1320 | 1345 |  | 1414 | 1311 | 1328 |
| 20 | 1186 | 1275 | 1240 | 1226 | 1201 | 1183 | 1166 | 1211 | 1240 | 1260 | 1211 | 1171 | 1206 | 1214 | 1228 | 1230 | 1234 | 1225 |
| 21 | 1195 | 1255 | 1212 | 1167 | 1180 | 1237 | 1204 | 1192 | 1189 | 1194 | 1132 | 1187 | 1110 | 1174 | 1183 | 1139 | 1113 | 1163 |
| 22 | 1175 | 1230 | 1256 | 1300 | 1251 | 1177 | 1106 | 1231 | 1238 | 1253 | 1204 | 1194 | 1222 | 1218 | 1225 | 1185 | 1165 | 1235 |
| 23 | 1168 | 1279 | 1240 | 1224 | 1208 | 1184 | 1159 | 1211 | 1234 | 1245 | 1211 | 1151 | 1203 | 1213 | 1222 | 1222 | 1230 | 1233 |
| 24 | 1294 | 1337 | 1306 | 1360 | 1364 | 1293 | 1309 | 1328 | 1322 | 1330 | 1294 | 1310 | 1324 | 1326 | 1316 | 1324 | 1347 | 1340 |
| 25 | 1274 | 1300 | 1280 | 1315 | 1356 | 1202 | 1236 | 1259 | 1250 | 1231 | 1201 | 1240 | 1185 | 1255 | 1256 | 1207 | 1199 | 1219 |
| 26 | 1166 | 1276 | 1247 | 1227 | 1206 | 1184 | 1161 | 1213 | 1240 | 1240 | 1210 | 1162 | 1219 | 1214 | 1224 | 1240 | 1235 | 1234 |
| 27 | 1262 | 1305 | 1313 | 1299 | 1243 | 1227 | 1302 | 1323 | 1362 | 1296 | 1241 | 1260 | 1284 | 1311 | 1340 | 1273 | 1219 | 1269 |
| 28 | 1181 | 1235 | 1235 | 1219 | 1174 | 1187 | 1188 | 1239 | 1214 | 1229 | 1224 | 1231 | 1195 | 1223 | 1230 | 1220 | 1226 | 1211 |
| 29 | 1265 | 1247 | 1181 | 1224 | 1276 | 1192 | 1229 | 1229 | 1155 | 1198 | 1234 | 1247 | 1371 | 1287 | 1249 | 1155 | 1267 | 1328 |
| 30 | 1209 | 1242 | 1250 | 1266 | 1211 | 1177 | 1192 | 1267 | 1295 | 1255 | 1200 | 1228 | 1209 | 1278 | 1277 | 1256 | 1274 | 1267 |
| 31 | 1224 | 1253 | 1299 | 1263 | 1203 | 1146 | 1253 | 1218 | 1261 | 1306 | 1230 | 1236 | 1226 | 1220 | 1275 | 1250 | 1256 | 1201 |
| 32 | 1189 | 1191 | 1205 | 1221 | 1210 | 1203 | 1191 | 1205 | 1200 | 1229 | 1208 | 1178 | 1185 | 1178 | 1187 | 1201 | 1196 | 1196 |
| 33 | 1626 | 1254 | 1220 | 1409 | 1431 | 1348 | 1317 | 1243 | 1207 | 1237 | 1301 | 1245 | 1219 | 1203 | 1186 | 1205 | 1209 | 1241 |
| 34 | 1243 | 1240 | 1333 | 1321 | 1190 | 1222 | 1246 | 1260 | 1263 | 1205 | 1227 | 1245 | 1296 | 1217 | 1279 | 1361 | 1270 | 1283 |
| 35 | 1094 | 1170 | 1185 | 1219 | 1207 | 1128 | 1117 | 1172 | 1211 | 1271 | 1221 | 1167 | 1190 | 1184 | 1253 | 1281 | 1242 | 1228 |
| 36 | 1239 | 1205 | 1149 | 1188 | 1182 | 1169 | 1206 | 1208 | 1186 | 1222 | 1222 | 1176 | 1170 | 1195 | 1198 | 1217 | 1226 | 1219 |
| 37 | 1176 | 1278 | 1249 | 1224 | 1206 | 1185 | 1160 | 1215 | 1238 | 1241 | 1211 | 1159 | 1219 | 1216 | 1227 | 1239 | 1234 | 1233 |
| 38 | 1237 | 1218 | 1237 | 1218 | 1193 | 1216 |  | 1270 | 1434 | 1430 | 1382 |  | 1244 | 1256 | 1357 | 1275 | 1230 |  |
| 39 | 1230 | 1219 | 1220 | 1209 | 1213 | 1236 | 1211 | 1247 | 1240 | 1258 | 1257 | 1207 | 1250 | 1260 | 1212 | 1274 | 1233 | 1259 |
| 40 | 1190 | 1191 | 1206 | 1219 | 1215 | 1204 | 1190 | 1205 | 1196 | 1231 | 1210 | 1176 | 1185 | 1170 | 1192 | 1202 | 1199 | 1197 |
| 41 | 1206 | 1242 | 1250 | 1260 | 1211 | 1177 | 1190 | 1266 | 1285 | 1255 | 1201 | 1227 | 1210 | 1271 | 1277 | 1254 | 1269 | 1266 |
| 42 | 1231 | 1222 | 1216 | 1210 | 1212 | 1233 | 1214 | 1252 | 1244 | 1250 | 1254 | 1215 | 1254 | 1260 | 1211 | 1301 | 1232 | 1259 |
| 43 | 1281 | 1282 | 1283 | 1328 | 1248 | 1258 | 1251 | 1260 | 1289 | 1290 | 1213 | 1260 | 1212 | 1253 | 1259 | 1288 | 1259 | 1250 |
| 44 | 1223 | 1253 | 1311 | 1265 | 1199 | 1147 | 1257 | 1222 | 1267 | 1306 | 1234 | 1231 | 1230 | 1221 | 1275 | 1257 | 1260 | 1200 |
| 45 | 1166 | 1274 | 1240 | 1220 | 1205 | 1184 | 1160 | 1212 | 1238 | 1242 | 1200 | 1169 | 1218 | 1212 | 1223 | 1230 | 1233 | 1234 |
| 46 | 1264 | 1246 | 1182 | 1225 | 1276 | 1190 | 1229 | 1230 | 1150 | 1200 | 1228 | 1250 | 1370 | 1287 | 1249 | 1145 | 1266 | 1329 |
| 47 | 1261 | 1298 | 1337 | 1314 | 1256 | 1238 | 1278 | 1327 | 1399 | 1363 | 1312 | 1313 | 1316 | 1355 | 1480 | 1414 | 1315 | 1320 |
| 48 | 1385 | 1452 | 1388 | 1382 | 1375 | 1359 | 1330 | 1347 | 1350 | 1297 | 1342 | 1256 | 1355 | 1366 | 1334 | 1275 | 1351 | 1300 |
| 49 | 1202 | 1301 | 1276 | 1240 | 1273 | 1196 | 1407 | 1355 | 1457 | 1313 | 1226 | 1325 | 1240 | 1319 | 1306 | 1258 | 1317 | 1257 |
| 50 | 1195 | 1274 | 1278 | 1293 | 1266 | 1262 | 1418 | 1346 | 1325 | 1360 | 1332 | 1344 | 1215 | 1184 | 1266 | 1214 | 1306 | 1161 |
| 51 | 1282 | 1280 | 1263 | 1300 | 1251 | 1285 | 1194 | 1275 | 1276 | 1341 | 1340 | 1304 | 1326 | 1270 | 1328 | 1341 | 1291 | 1304 |
| 52 | 1183 | 1226 | 1243 | 1283 | 1211 | 1164 | 1184 | 1225 | 1264 | 1264 | 1323 | 1167 | 1184 | 1278 | 1258 | 1258 | 1179 | 1169 |
| 53 | 1244 | 1253 | 1334 | 1320 | 1194 | 1224 | 1248 | 1264 | 1263 | 1206 | 1225 | 1245 | 1295 | 1218 | 1280 | 1360 | 1270 | 1282 |
| 54 | 1394 | 1541 | 1245 | 1101 | 1538 | 1525 | 1518 | 1294 | 1406 | 1412 | 1282 | 1360 | 1226 | 1290 | 1288 | 1219 | 1347 | 1199 |
| 55 | 1129 | 1307 | 1300 | 1288 | 1294 | 1214 | 1340 | 1317 | 1389 | 1332 | 1298 | 1424 | 1289 | 1353 | 1432 | 1303 | 1412 | 1505 |
| 56 | 1200 | 1302 | 1277 | 1237 | 1270 | 1192 | 1407 | 1350 | 1452 | 1313 | 1224 | 1324 | 1238 | 1318 | 1304 | 1257 | 1317 | 1255 |
| 57 | 1325 | 1328 | 1350 | 1378 | 1306 | 1279 | 1237 | 1265 | 1302 | 1389 | 1349 | 1277 | 1283 | 1229 | 1245 | 1249 | 1379 | 1304 |
| 58 | 1215 | 1320 | 1319 | 1320 | 1369 | 1289 | 1220 | 1267 | 1279 | 1236 | 1247 | 1175 | 1140 | 1239 | 1262 | 1280 | 1284 | 1217 |
| 59 | 1274 | 1306 | 1282 | 1316 | 1355 | 1205 | 1238 | 1266 | 1256 | 1236 | 1208 | 1239 | 1191 | 1256 | 1252 | 1219 | 1197 | 1220 |
| 60 | 1166 | 1247 | 1272 | 1296 | 1336 | 1218 | 1101 | 1205 | 1243 | 1248 | 1249 | 1175 | 1090 | 1159 | 1247 | 1302 | 1238 | 1261 |
| 61 | 1205 | 1318 | 1321 | 1319 | 1368 | 1289 | 1217 | 1269 | 1275 | 1232 | 1249 | 1175 | 1141 | 1239 | 1262 | 1284 | 1283 | 1210 |
| 62 | 1159 | 1257 | 1240 | 1276 | 1220 | 1193 | 1129 | 1170 | 1190 | 1205 | 1156 | 1180 | 1134 | 1190 | 1197 | 1216 | 1211 | 1162 |
| 63 | 1329 | 1316 | 1314 | 1350 | 1298 | 1312 | 1383 | 1304 | 1308 | 1364 | 1254 | 1349 | 1393 | 1333 | 1387 | 1497 | 1331 | 1335 |
| 64 | 1324 | 1325 | 1350 | 1376 | 1312 | 1279 | 1230 | 1266 | 1302 | 1388 | 1347 | 1273 | 1283 | 1228 | 1239 | 1249 | 1365 | 1304 |
| 65 | 1263 | 1272 | 1293 | 1281 | 1223 | 1287 | 1277 | 1284 | 1248 | 1299 | 1240 | 1393 | 1284 | 1315 | 1322 | 1228 | 1210 | 1312 |
| 66 | 1300 | 1372 | 1229 | 1243 | 1290 | 1263 | 1213 | 1348 | 1296 | 1227 | 1156 | 1120 | 1220 | 1327 | 1310 | 1261 | 1323 | 1295 |
| 67 | 1216 | 1319 | 1321 | 1321 | 1373 | 1288 | 1218 | 1269 | 1277 | 1238 | 1247 | 1176 | 1140 | 1240 | 1262 | 1285 | 1284 | 1216 |
| 68 | 1162 | 1218 | 1234 | 1254 | 1248 | 1178 | 1156 | 1233 | 1235 | 1242 | 1198 | 1174 | 1207 | 1215 | 1210 | 1185 | 1159 | 1235 |
| 69 | 1231 | 1307 | 1297 | 1288 | 1294 | 1214 | 1340 | 1317 | 1389 | 1332 | 1298 | 1310 | 1298 | 1353 | 1432 | 1323 | 1412 | 1392 |
| 70 | 1229 | 1230 | 1222 | 1211 | 1212 | 1235 | 1232 | 1264 | 1250 | 1258 | 1260 | 1208 | 1256 | 1262 | 1260 | 1302 | 1236 | 1259 |
| 71 | 1269 | 1220 | 1267 | 1289 | 1222 | 1301 | 1290 | 1209 | 1294 | 1259 | 1229 | 1204 | 1210 | 1235 | 1291 | 1265 | 1247 | 1211 |
| 72 | 1251 | 1348 | 1288 | 1304 | 1315 | 1264 | 1297 | 1278 | 1291 | 1395 | 1313 | 1257 | 1270 | 1245 | 1293 | 1309 | 1275 | 1275 |
| 73 | 1275 | 1305 | 1285 | 1317 | 1356 | 1203 | 1236 | 1268 | 1258 | 1232 | 1207 | 1232 | 1192 | 1256 | 1254 | 1223 | 1199 | 1222 |
| 74 | 1267 | 1257 | 1301 | 1272 | 1234 | 1204 | 1204 | 1202 | 1247 | 1314 | 1231 | 1224 | 1204 | 1173 | 1420 | 1403 | 1225 | 1235 |
| 75 | 1157 | 1213 | 1226 | 1157 | 1180 | 1199 | 1151 | 1206 | 1228 | 1211 | 1204 | 1173 | 1140 | 1182 | 1211 | 1206 | 1170 | 1179 |
| 76 | 1290 | 1357 | 1392 | 1370 | 1378 | 1331 | 1310 | 1325 | 1347 | 1318 | 1388 | 1339 | 1331 | 1349 | 1291 | 1200 | 1360 | 1357 |
| 77 | 1303 | 1373 | 1230 | 1240 | 1291 | 1263 | 1210 | 1350 | 1296 | 1212 | 1149 | 1123 | 1218 | 1328 | 1310 | 1267 | 1354 | 1301 |
| 78 | 1172 | 1231 | 1261 | 1308 | 1247 | 1178 | 1097 | 1232 | 1238 | 1243 | 1210 | 1186 | 1225 | 1219 | 1225 | 1195 | 1165 | 1236 |
| 79 | 1223 | 1332 | 1272 | 1271 | 1280 | 1211 | 1249 | 1332 | 1269 | 1272 | 1254 |  |  | 1333 | 1311 | 1352 | 1332 |  |
| 80 | 1230 | 1227 |  | 1294 | 1261 | 1212 | 1177 | 1224 | 1238 | 1261 | 1240 | 1247 | 1205 | 1280 | 1281 | 1275 | 1238 | 1219 |
